# A global model-agnostic rule-based XAI method based on Parameterized Event Primitives for time series classifiers

**DOI:** 10.3389/frai.2024.1381921

**Published:** 2024-09-20

**Authors:** Ephrem Tibebe Mekonnen, Luca Longo, Pierpaolo Dondio

**Affiliations:** ^1^School of Computer Science, College of Health and Science, Technological University Dublin, Dublin, Ireland; ^2^Artificial Intelligence and Cognitive Load Research Lab, Technological University Dublin, Dublin, Ireland

**Keywords:** deep learning, Explainable Artificial Intelligence, time series classification, decision tree, model agnostic, *post-hoc*

## Abstract

Time series classification is a challenging research area where machine learning and deep learning techniques have shown remarkable performance. However, often, these are seen as black boxes due to their minimal interpretability. On the one hand, there is a plethora of eXplainable AI (XAI) methods designed to elucidate the functioning of models trained on image and tabular data. On the other hand, adapting these methods to explain deep learning-based time series classifiers may not be straightforward due to the temporal nature of time series data. This research proposes a novel global *post-hoc* explainable method for unearthing the key time steps behind the inferences made by deep learning-based time series classifiers. This novel approach generates a decision tree graph, a specific set of rules, that can be seen as explanations, potentially enhancing interpretability. The methodology involves two major phases: (1) training and evaluating deep-learning-based time series classification models, and (2) extracting parameterized primitive events, such as increasing, decreasing, local max and local min, from each instance of the evaluation set and clustering such events to extract prototypical ones. These prototypical primitive events are then used as input to a decision-tree classifier trained to fit the model predictions of the test set rather than the ground truth data. Experiments were conducted on diverse real-world datasets sourced from the UCR archive, employing metrics such as accuracy, fidelity, robustness, number of nodes, and depth of the extracted rules. The findings indicate that this global *post-hoc* method can improve the global interpretability of complex time series classification models.

## 1 Introduction

Due to the affordability of sensors, time series data have become prevalent in various domains, including finance (Zhang et al., 2019), healthcare (Liu et al., 2022; Strodthoff et al., 2020), recognition of human activity (Mekruksavanich and Jitpattanakul, 2021; Joshi and Abdelfattah, 2021), and environmental monitoring (Shu et al., 2019). Time series classification involves categorizing or assigning a class label to a given time series, a critical task in scenarios where sensor or financial data analysis is essential for informed business decisions. Various algorithms have been devised for time series classification. Deep learning models have shown exceptional effectiveness in tasks such as computer vision, natural language processing, and time series classification. However, these models are often deemed opaque due to their complex architecture and lack of transparency, giving rise to research in Explainable Artificial Intelligence (XAI) to address this limitation (Longo et al., 2023). XAI is a growing field of research that aims to address this issue by (i) developing techniques that aim at providing understandable and transparent explanations of machine learning models and (ii) evaluating and assessing their impact on humans (Theissler et al., 2022; Di Martino and Delmastro, 2022; Vilone and Longo, 2023). Several XAI methods have been proposed for deep learning-based time series classification models to overcome these issues. These techniques include using commonly used XAI methods for computer vision (Schlegel et al., 2019), such as Local Interpretable Model-agnostic Explanations (LIME)(Ribeiro et al., 2016), Saliency Maps (Simonyan et al., 2013), and Layer-wise Relevance Propagation (LRP)(Bach et al., 2015).

However, adapting existing XAI methods for image and tabular data to time series data presents unique challenges due to the need to account for the temporal nature of the data (Schlegel et al., 2019; Theissler et al., 2022). These methods often produce heatmap-based explanations that are hard to interpret and primarily developer-focused (Rojat et al., 2021; Jeyakumar et al., 2020). Moreover, feature importance methods such as bespoke LIME and SHAP fail to capture temporal dependencies by treating each time step or segment independently.

This research addresses these limitations by offering global rule-based explanations using parameterized event primitives, which represent specific types of events such as increasing or decreasing trends, local maxima, and local minima. These parameterized events effectively capture and convey inherent temporal patterns, making explanations more intuitive and comprehensible (Kadous, 1999). The approach generates a decision tree that provides a set of rules assumed to be more understandable to humans, making it easier for non-experts to comprehend a model's predictions. Decision trees are considered interpretable by design and can provide insights into the relationships between features and the output (Molnar, 2020). Furthermore, they can be easily visualized, facilitating comprehension of inference chains (Vilone and Longo, 2023).

The main contribution of this research is a novel global *post-hoc* XAI method to explain the inference process of deep learning-based time series classification models using a decision tree based on parameterized event primitives.

Finally, we point out that our approach is a good starting point for further improvement and the generation of explanatory descriptions that back up AI decisions of time series classification models.

The rest of the paper is structured as follows: Section 2 reviews existing XAI methods that have been used to explain deep learning-based time series classifiers. Section 3 outlines the proposed approach. In Section 4, the experimental results are presented and discussed in detail. Finally, Section 5 concludes the article and highlights possible future directions.

## 2 Related work

In recent years, the surge of interest in Explainable Artificial Intelligence (XAI) methods has gained attention to address the transparency and interpretability challenges posed by complex models within the field of machine learning. In particular, two pivotal paradigms within the XAI framework are attributions and attentions (Theissler et al., 2022).

Attribution methods, encompassing techniques such as LIME (Ribeiro et al., 2016), Saliency Maps (Simonyan et al., 2013), SHAP (Lundberg and Lee, 2017), and LRP (Bach et al., 2015), have played a critical role in computer vision for elucidating salient features within input data. The application of these methods has seamlessly transitioned to the domain of time series analysis, as evidenced by the works of Schlegel et al. (2019), particularly the work described in Neves et al. (2021) and Sivill and Flach (2022), adapted LIME for direct application to time series data. Advancing the discourse on time series classifiers, Zhou et al. (2021) have enriched the interpretability landscape by enhancing Class Activation Maps (CAM) and Grand-CAM with backpropagation. Simultaneously, the work described in Siddiqui et al. (2019) introduced TSViz, a saliency map-based methodology later integrated into TSXplain (Munir et al., 2019) for unearthing the logic behind Deep Neural Networks (DNNs) in time series. These methodologies combine salient regions, instances, and statistical features, thereby fostering natural language explanations.

In the realm of time series data, Vielhaben et al. (2023) have introduced DFT-LRP, a tailored variant of Layer-wise Relevance Propagation (LRP). This methodology is purposefully designed to cater to the intricacies of time series data and involves the incorporation of a virtual inspection layer preceding the input layer, an innovative step facilitating the transformation of time series data and enabling the propagation of relevance attributions through Layer-wise Relevance Propagation (LRP).

Despite the efficacy of attributions, their application to time series data is not without challenges, due to the non-intelligible nature of time series (Schlegel and Keim, 2021). Heat maps, often used in the visualization of attributions, are promising for domain experts, but pose challenges for general users (Jeyakumar et al., 2020). Additionally, the assumption of feature independence inherent in attributions is frequently violated when considering adjacent observations within time series data (Watson, 2022).

Similarly, attention mechanisms, notably exemplified by Karim et al. (2017), share a challenge in visual interpretation similar to that faced by attribution methods, often relying on heatmaps.

In the midst of the prevailing emphasis on local interpretability in XAI research, particularly in time series data, it is crucial to recognize researchers contributing to global insights in time series classifiers. The work described in Oviedo et al. (2019) generalizes CAM to encompass all instances within a class, offering an average CAM for comprehensive insight. Moreover, Siddiqui et al. (2019) focus on clustering filters, while Cho et al. (2020) concentrate on clustering input sequences, both enriching global understanding through grouping based on activation patterns.

Despite the multitude of Explainable Artificial Intelligence (XAI) methods dedicated to explaining specific instances in time series data, there is a noticeable gap. There is a lack of methods not tied to a specific model and can easily provide comprehensive global insights. Our novel approach presents a global model-agnostic method to explain deep learning-based time series classifiers using a decision tree. This approach aims to maintain the temporal dependency inherent in time-series data while providing explanations in an understandable format.

Our methodology falls within the domain of surrogate-based approaches, as we leverage linear models like decision trees to mimic the inference process of deep learning time series classifiers. The method produces a set of rules or a decision tree graph as an explanation, making it transparent and easy to comprehend. Decision tree-based explanations are intuitive and structured, representing the logic of an ML model as a set of rules that can be easily interpreted and visualized. Therefore, they are considered naturally transparent and intelligible by scholars (Vilone et al., 2020).

## 3 Proposed method

**Figure 1:**
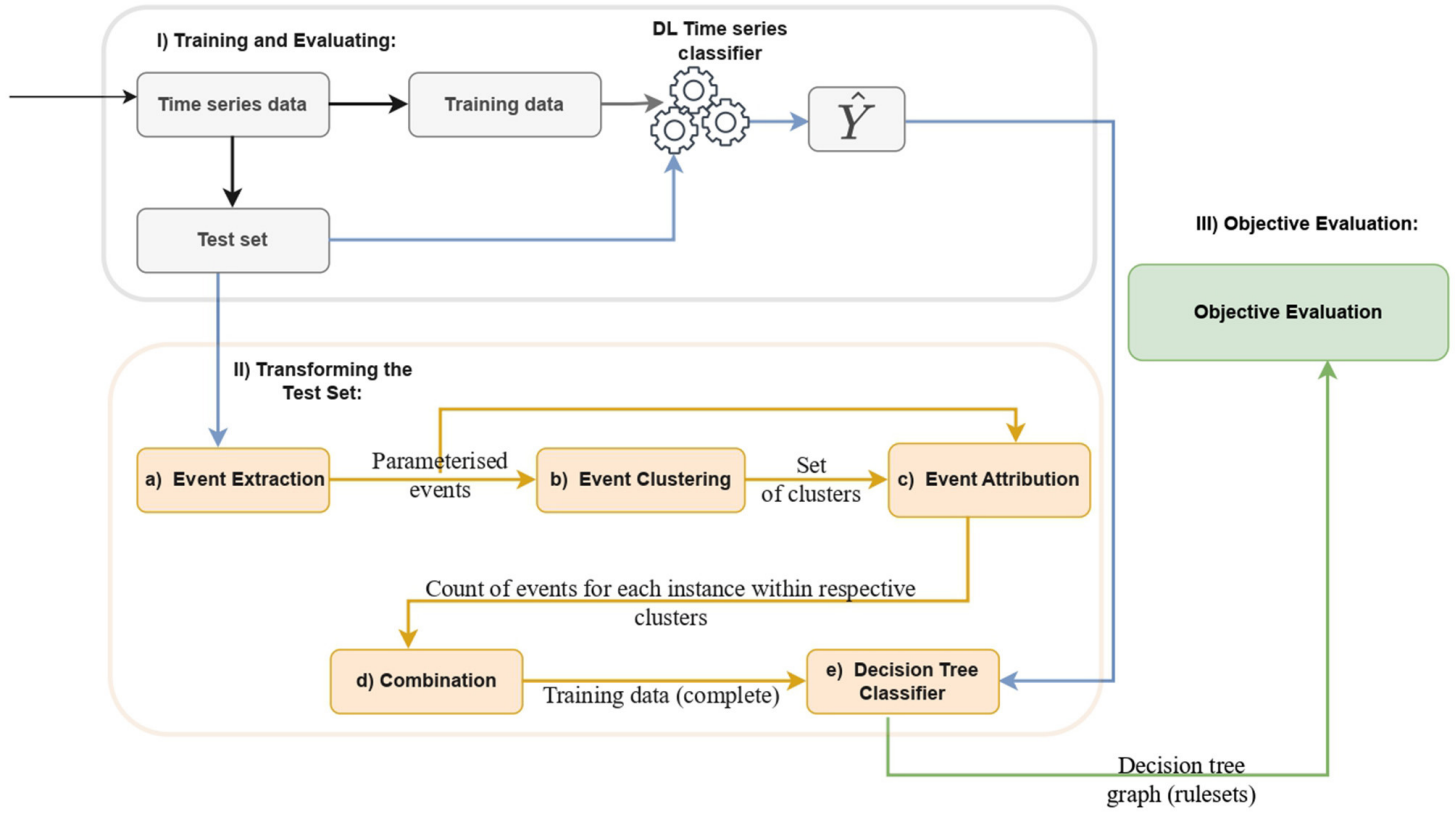
Design of the proposed method: (Phase I) Initial data preprocessing for training and evaluating a deep learning-based time series classifier. (Phase II) Sub-steps include **(a)** Extraction of Parameterized Event Primitives (PEPs) from the test set, encompassing events like increasing, decreasing, flat, local maximum, and local minimum. **(b)** Clustering of PEPs, **(c)** Event attribution by counting events belonging to each cluster using the extracted events and predefined clusters, **(d)** Concatenating each data frame of PEPs produced during the event attribution step, and **(e)** Training and testing of the decision tree using the transformed test set and the model prediction. (Phase III) Evaluation of decision tree rules using objective metrics, including accuracy, fidelity, complexity, and robustness.

This section introduces a novel model-agnostic *post-hoc* Explainable Artificial Intelligence (XAI) method for deep learning-based time series classifiers. Figure 1 illustrates the diagram of our proposed method, which consists of three distinct phases. In what follows, we provide a detailed explanation of the method.

### 3.1 Phase I: training and evaluating

The initial phase of the method involves preparing the data and subsequently training and evaluating the targeted deep-learning models for explanation.

### 3.2 Phase II: transforming the test set

Parameterized Event Primitives (PEPs) are extracted from the test set of the deep learning model as shown in Figure 1. Parameterized Event Primitives (PEPs) are enlisted to extract events defined by a tuple of parameters and a finding function presumed to manifest in the domain. Extracting PEPs from a time series helps to represent the temporal characteristics of events as parameters, which facilitates learning for interpretable models such as decision trees (Kadous, 1999). In this study, an event refers to a specific pattern or behavior that is expected to occur in the domain. These events are defined using Parameterized Event Primitives such as increasing or decreasing trends, local maxima, and local minima, which are intuitive and meaningful to users.

The methodology outlined in Kadous (1999) is implemented with a modification (in Subsection 3.2.3 at the event attribution stage) that aims to count the number of events within a cluster (event_cluster_num_), rather than simply indicating their presence or absence with a binary representation. This refinement contributes to a noticeable improvement in the decision tree performance, particularly manifesting significant improvements in specific datasets. The accuracy of the decision tree assumes paramount importance, given its consequential impact on the enhancement of fidelity. Subsequently, the sections detail each step for transforming the test data to train and evaluate the surrogate decision tree.

#### 3.2.1 Extracting Parameterized Event Primitives

In this step, we extract Parameterized Event Primitives (PEPs) from each evaluation set time series sequence. Let a time series sequence be denoted as *x* = *x*_1_, *x*_2_, …, *x*_*n*_, where *x*_*i*_ represents the time series value at time *i*. The function that extracts the events takes a series as input and returns a list of extracted events, denoted as E. For example, considering the increasing event, *E*_*inc*_ can be represented as the set of tuples where each tuple contains the time when a positive gradient begins (*t*_start_), the duration until the gradient stops increasing (dura), and the average gradient values (*grad*_avg_). This can be formally denoted as:


$$E_{\text{inc}}=\left\{\begin{array}{cc} \left(t_{{\text{start}}_{1}},{\text{dura}}_{1},{\text{grad}}_{{\text{avg}}_{1}}\right),\\ \left(t_{{\text{start}}_{2}},{\text{dura}}_{2},{\text{grad}}_{{\text{avg}}_{2}}\right), & \ldots \end{array}\right\}$$

**Figure 2:**
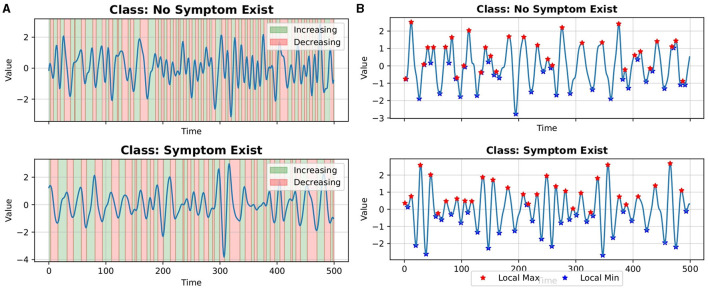
Examples of events extracted from a single time series **(A)** increasing and decreasing events **(B)** local max and local min events.

**Figure 3:**
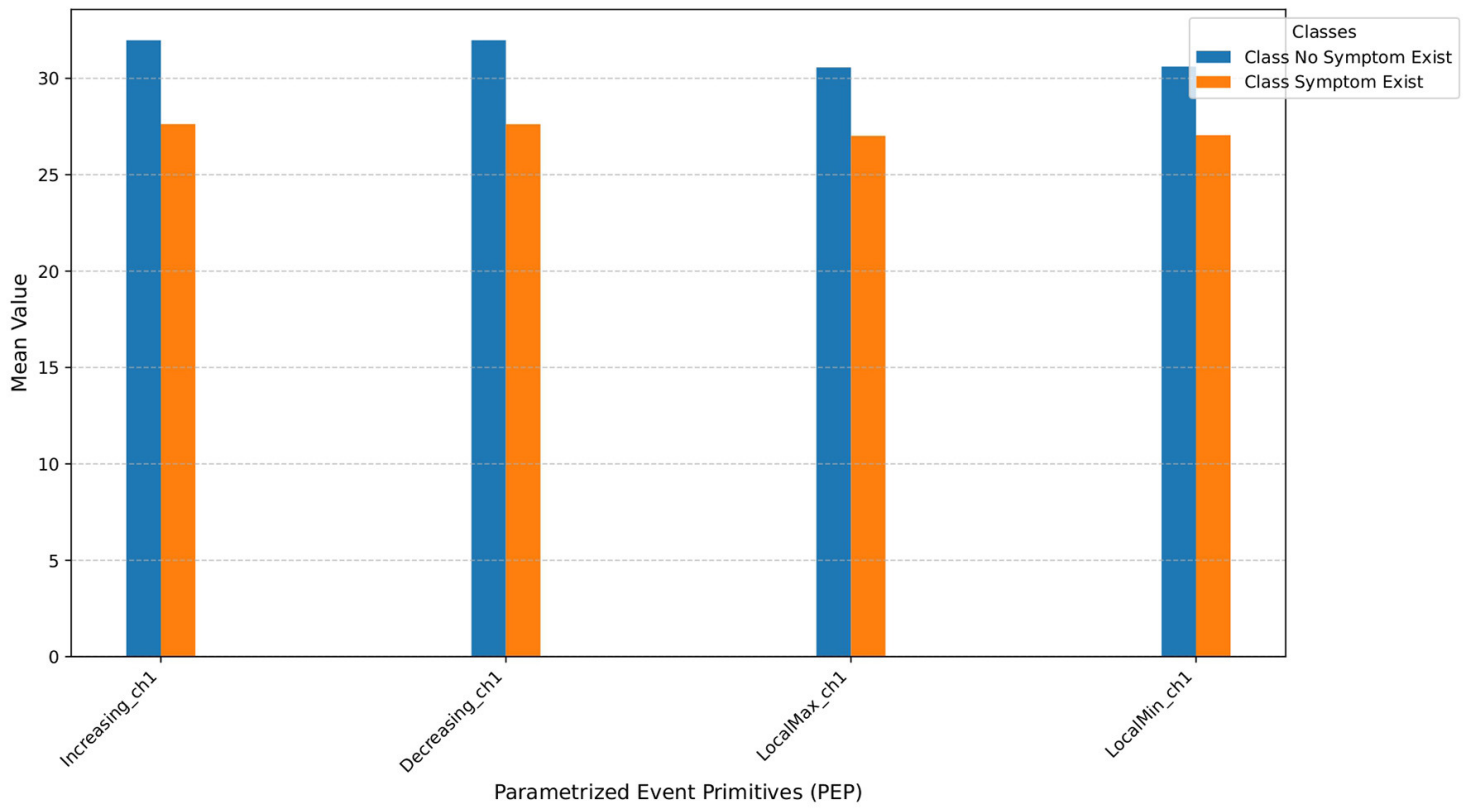
Average number of extracted events for each parameterized event primitives on Ford A dataset. The ‘_ch1' suffix denotes the channel number; in this context, it signifies a univariate time series due to a single channel.

Figures 2A, B show examples of extracted events from a single time series. Figure 3 shows the average number of extracted events per class for each parameterized event primitive across the entire Ford A dataset evaluation set.

#### 3.2.2 Event clustering

**Figure 4:**
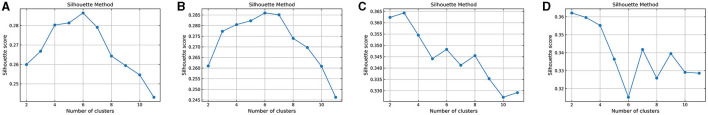
Optimal number of clusters obtained using silhouette method for **(A)** increasing events, **(B)** decreasing events, **(C)** local max events, and **(D)** local min events, of FordA data.

Each parameterized event, denoted as *E*, undergoes a flattening process to apply a clustering algorithm (for instance, flattening increasing events across all the test set cases). The KMeans clustering algorithm was used in this experiment, with the silhouette method determining the optimal number of clusters. The optimal number corresponds to the highest average silhouette score, as illustrated in Figure 4.

**Figure 5:**
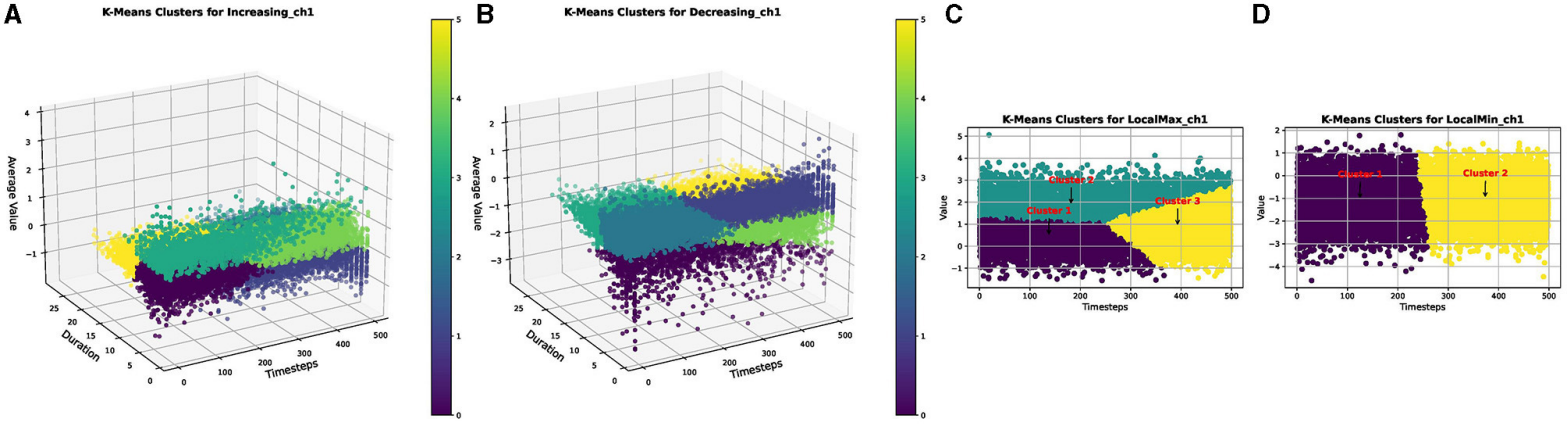
Clusters produced by KMeans for **(A)** increasing events, **(B)** decreasing events, **(C)** local maxima events, and **(D)** local minima events, of the FordA dataset.

This iterative procedure is executed for all four extracted parameterized events: increasing events (*E*_inc_), decreasing events (*E*_dec_), local maxima events (*E*_max_), and local minima events (*E*_min_). Figure 5 visually represents a set of clusters generated by the clustering algorithms for each parameterized event.

Each cluster, denoted as *C*_*i, j*_, where *i* signifies the type of parameterized event and *j* represents the cluster index, serves as the foundation unit for the subsequent event attribution step.

#### 3.2.3 Event attribution

At this step, the extracted events *E* and the set of clusters *C*_*j*_ are taken as input. The output of this process is a data frame *D*, where instances are represented along rows and clusters along columns. Each cell *D*_*i, j*_ denotes the number of events from the extracted set of events belonging to each cluster for a specific instance:


$$D_{i,j}=\overset{n}{\underset{k=0}{\sum }}I\left(E_{i,k}\in C_{j}\right)$$

**Figure 6:**
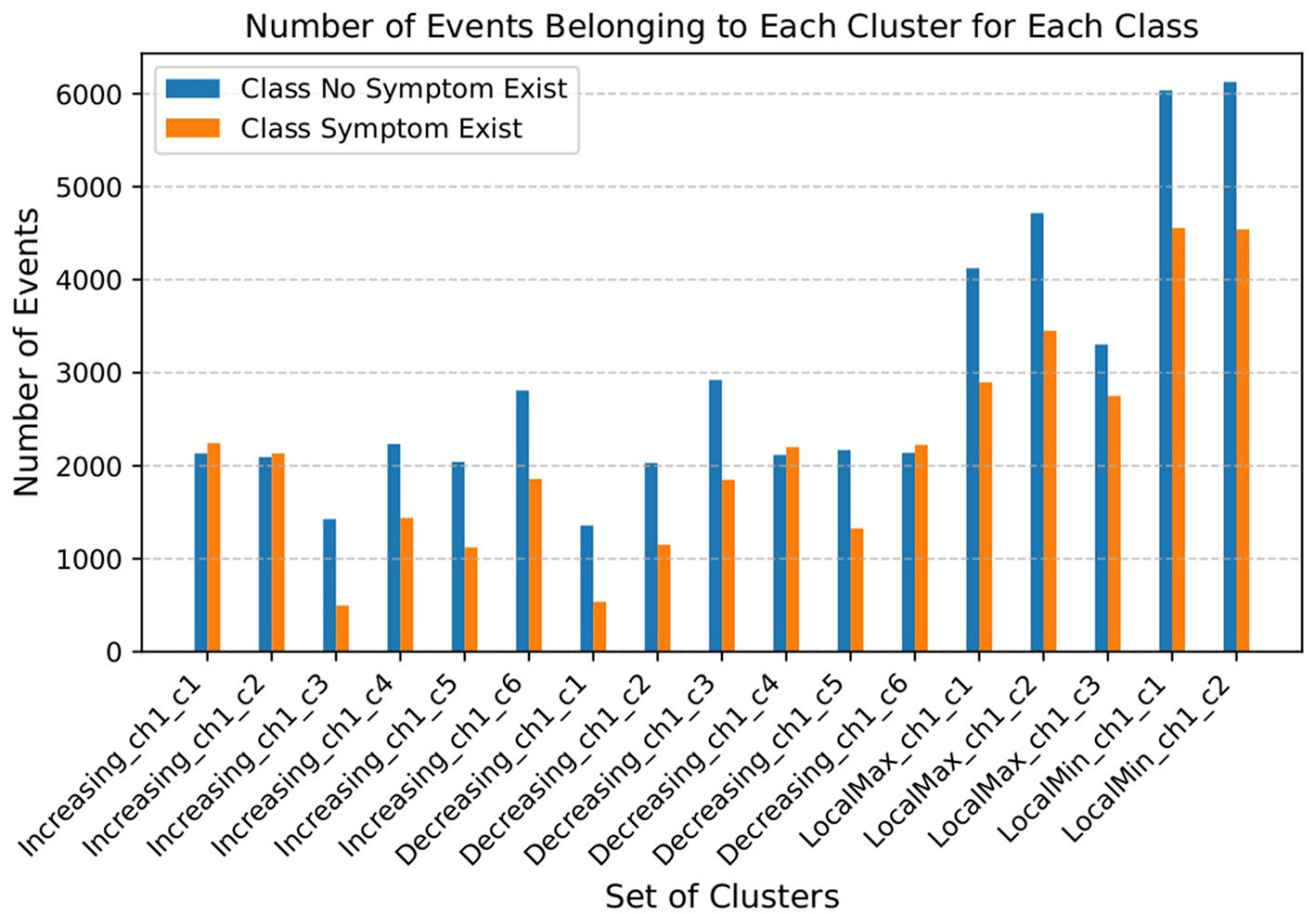
Number of events belonging to each parameterized event cluster.

Here, *k* represents the index of the event in the list of the extracted events of *i* instance of the dataset, and *n* represents the length of the event (*i*.*e*., *n* = len(*E*)), and *I*(·) is the indicator function that equals 1 if the condition inside the parentheses is true and 0 otherwise. Figure 6 depicts the average number of events in each cluster within the event *E*.

#### 3.2.4 Combination

Following the event attribution step, the resultant data frames corresponding to each parameterized event are combined to construct the training data set for the decision tree classifier. Upon the culmination of this process, a comprehensive training dataset is acquired and employed to train the decision tree classifier.

#### 3.2.5 Train decision tree classifier

After transforming the test set, the next step is to apply the decision tree classifier. To do this, we split the transformed data into training and testing sets, with 70% of the data used for training and 30% used for testing.

### 3.3 Phase III: objective evaluation

To objectively and quantitatively assess the interpretability of our method, we selected five metrics: accuracy, fidelity, robustness, depth, and number of nodes. To achieve objectivity, we exclude any human intervention in the evaluation process. Accuracy measures the fraction of correct predictions made by the model, while fidelity evaluates the consistency between the model's decision and the explanation provided by the decision tree. Depth and number of nodes measure the complexity of the decision tree. Robustness measures the XAI method's resilience to minor input changes that do not affect the model predictions. Refer to Table 1 for an in-depth presentation of the objective evaluation metrics.

**Table 1:** Objective evaluation metrics for rule-based explanation.

**Metric**	**Definition**	**Formula**
Accuracy	The proportion of correctly predicted instances (*c*) out of the total instances (*N*).	$\text{A}=\frac{\text{c}}{\text{N}}$
Fidelity	Ratio of input instances where the surrogate model agrees (a) with the actual model, divided by the total number of instances (N)	$\text{F}=\frac{\text{a}}{\text{N}}$
Complexity	The complexity or simplicity of the generated explanation is measured by the number of nodes and depth	C = # Depth, #Nodes
Robustness	The persistence of methods (the surrogate model (*g*(*x*_*n*_)) in our case) to withstand small perturbations (δ) of the input that does not change the prediction of the model (*f*(*x*_*n*_)).	$\text{R}=\frac{\overset{N}{\underset{n=1}{\sum }}\left[g\left(x_{n}\right)=g\left(x_{n}+\delta \right)\right]}{N}$

## 4 Experimental settings

### 4.1 Datasets and models

We specifically chose four univariate time series datasets (ECG 200, Gunpoint, FordA, and FordB) from the 2018 UCR archive to assess the effectiveness of our proposed method. The ECG200 dataset comprises a set of time series. Each series traces the electrical activity recorded during one heartbeat. The dataset has two classes: normal heartbeat and myocardial infarction. The GunPoint time series dataset is a widely used benchmark for evaluating the performance of time series classification algorithms. It consists of 200 univariate time series representing hand movement trajectories of one male and one female actor to classify hand movement into point gesture and gun gesture. The FordA and FordB datasets contain time series data of engine noise collected during standard operating conditions to classify the presence or absence of symptoms. However, the FordB dataset is gathered in a noisy environment. Refer to Table 2 for detailed statistics on the datasets. In terms of class distribution, ECG200 displays a slight imbalance, with Class 0 comprising 67 instances and Class 1 comprising 133 instances, resulting in a ratio of ~2:1. In contrast, Gunpoint demonstrates a balanced distribution, with both classes containing 100 instances each. Similarly, FordA and FordB datasets maintain balanced distributions, with each maintaining a 1:1 ratio. FordA contains 2,527 instances in Class 0 and 2,394 instances in Class 1, while FordB showcases 2,261 instances in Class 0 and 2,185 instances in Class 1. Despite ECG200's slight imbalance, it does not significantly affect the analysis. To ensure consistent class distributions across training and test sets, we employed stratified splitting for all datasets. All datasets underwent minimal preprocessing, with batch-wise standardization applied before training using the TSStandardize() function from the tsai library.

**Table 2:** Statistics of four datasets used in the experiment.

**Name**	**Data size**	**No. classes**	**Length**
ECG 200	200	2	96
Gunpoint	200	2	150
Ford A	4,921	2	500
Ford B	4,446	2	500

We used two difficult-to-interpret architectures in our experimental setup: LSTM with a Fully Convolutional Network (LSTM-FCN) and a standalone Fully Convolutional Neural Network (FCN). These models were constructed using the PyTorch-based tsai library (Oguiza, 2023), with the current default configuration featuring kernel sizes of 7, 5, 3 for the convolutional layers and corresponding filter sizes of 128, 256, 128 specifically for the FCN. The FCN architecture comprises three one-dimensional convolutional layers, each integrated with batch normalization and ReLU activation, a Global Average Pooling (GAP) layer, and a softmax layer. The LSTM-FCN architecture combines Long Short Term Memory (LSTM) and Fully Convolutional Networks (FCN). The fully convolutional block consists of three stacked temporal convolutional blocks with filter sizes of 128, 256, and 128, respectively. The time series input is passed into the FCN and LSTM block. The output of the global pooling layer integrated at the end of FCN architecture and the LSTM block is concatenated and passed onto a softmax classification layer. Two models were trained and tested on the four selected datasets. The smaller datasets, ECG200 and Gunpoint, were partitioned into 60% for training, 15% for validation, and 25% for testing. The larger datasets, FordA and FordB, were partitioned into 70% for training, 15% for validation and 15% for testing. Both models demonstrated outstanding results. To prevent overfitting, early stopping was used during training with a patience of 15 and a minimum delta of 0.001. Furthermore, each model was trained 100 times using the Monte Carlo cross-validation technique with random training, validation, and test splits to ensure stable accuracy. The average performance of the models is presented in Table 3.

**Table 3:** Mean test and validation accuracy with standard deviation for FCN and LSTM-FCN models on four datasets.

**Dataset**	**FCN**		**LSTM FCN**	
	**Test Acc**	**Valid Acc**	**Test Acc**	**Valid Acc**
ECG200	0.87 ± 0.05	0.86 ± 0.07	0.86 ± 0.05	0.85 ± 0.05
GunPoint	0.99 ± 0.03	0.98 ± 0.07	0.98 ± 0.06	0.98 ± 0.07
FordA	0.90 ± 0.04	0.90 ± 0.04	0.91 ± 0.05	0.91 ± 0.05
FordB	0.88 ± 0.03	0.89 ± 0.04	0.86 ± 0.04	0.86 ± 0.04

### 4.2 Transforming the test set

After transformation, the test set used for evaluating the deep learning models, as explained in the Subsection 3.2, is employed to train and test the decision tree classifier to generate rules as an explanation. Unlike approaches focusing on local explanations for individual instances within the test set, our method provides a holistic understanding of the inference process of the black box model.

In this study, implemented PEPs include increasing and decreasing events, yielding three parameters (start time (start), duration (duration_event_), and the average value of the gradient (avg_gradient). Local max and local min events are also considered, providing two parameters (time of the maximum/minimum (time_max/min_) and the corresponding value (value_max/min_).

## 5 Result and discussion

**Figure 7:**
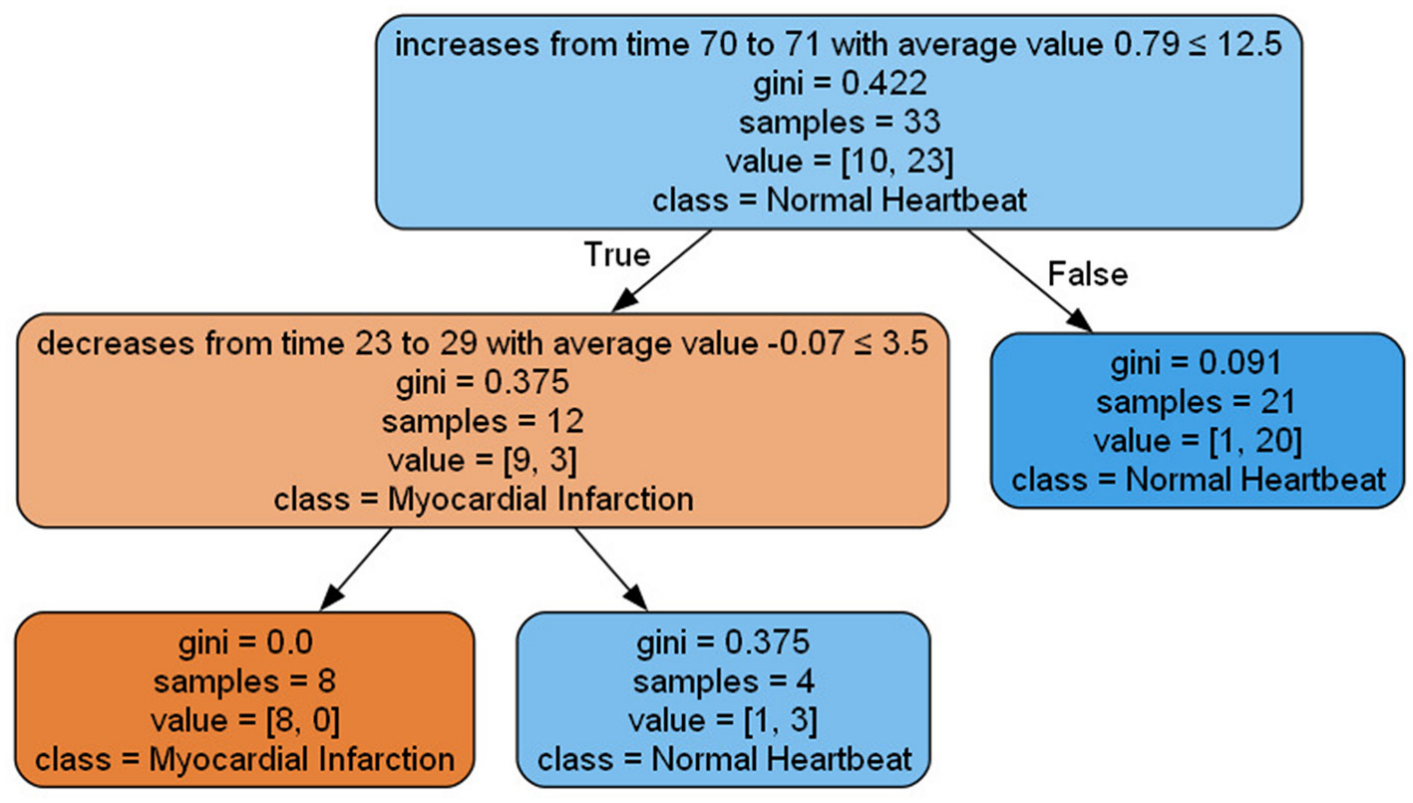
Visualization of decision tree graph produced by the proposed method applied to ECG data for the FCN model.

The objective evaluation results for the proposed XAI method are presented in Table 4, showcasing the mean and standard deviation of various objective evaluation metrics. The method was applied to four different datasets for two different models: Fully Convolutional Network (FCN) and LSTM FCN, and objectively evaluated using the metrics depicted in Table 1. Figure 7 illustrates the graph derived from a decision tree classifier trained on transformed data, as explained in Section 3C.

**Table 4:** Mean and standard deviation of the objective evaluation of the rule-based explanation.

**Dataset**	**FCN**					**LSTM FCN**				
	**Acc**	**Fidelity**	**#Depth**	**#Node**	**Rob**.	**Acc**	**Fidelity**	**#Depth**	**#Node**	**Rob**.
ECG200	0.79 ± 0.10	0.89 ± 0.06	3 ± 2	10 ± 6	0.78± 0.12	0.80 ± 0.12	0.89 ± 0.06	4 ± 2	10 ± 5	0.76 ± 0.14
GunPoint	0.74 ± 0.12	0.88 ± 0.11	4 ± 2	12 ± 5	0.64 ± 0.18	0.73 ± 0.11	0.88 ± 0.07	4 ± 2	12 ± 5	0.64 ± 0.17
FordA	0.78 ± 0.03	0.84 ± 0.04	8 ± 3	42 ± 34	0.76 ± 0.04	0.79 ± 0.04	0.84 ± 0.05	8 ± 4	41 ± 38	0.77 ± 0.05
FordB	0.81 ± 0.04	0.87 ± 0.05	8 ± 4	42 ± 34	0.77 ± 0.06	0.81 ± 0.04	0.86 ± 0.05	7 ± 4	37 ± 33	0.79 ± 0.08

For the FCN model, the accuracy values of the decision tree range from 0.74 to 0.81, reflecting how well the decision tree approximates the underlying complex model. Fidelity values, ranging from 0.84 to 0.89, indicate the agreement between the decision tree and the predictions of the deep learning model. The number of depth and nodes varies from 3 to 8 and 10 to 42, respectively, indicating the complexity of the decision tree graph or rules. The robustness scores range from 0.64 to 0.78, indicating its stability against insignificant data changes that do not affect model performance.

On the LSTM-FCN side, the accuracy values range from 0.73 to 0.81. The fidelity values, ranging from 0.84 to 0.89, indicate a high degree of alignment between the rule-based explanations and the predictions of the deep learning model. The LSTM-FCN model exhibits a more concise representation with a depth range of 4 to 8 and several nodes ranging from 10 to 41. The robustness scores for LSTM-FCN range from 0.64 to 0.79, almost similar to that of FCN. We fine-tuned the decision tree through a post-pruning technique, specifically using cost complexity pruning.

The list of rules extracted in the following demonstrates the findings of our experiment in the ECG200 data set. Each rule highlights the importance of particular time steps and the corresponding events occurring at those steps, significantly impacting the model prediction. Notably, the decision tree features represent clusters of each Parameterized Event Primitives (PEPs), and the nodes in the graph or rules symbolize the centroids of these clusters. For instance, let (*t, v*) represent the centroids obtained from Cluster 1 of local maxima. After post-processing, these centroids can be denoted as a Local Maximum event at time *t* with a value of *v*. It's important to note that for local maxima and local minima, the centroids consist of the variables time (*t*) and value (*v*). In the case of increasing and decreasing events, the centroids include time (*t*), duration (*d*), and average value ($\bar{v}$). Additionally, if domain experts provide definitions for the conditional part of the rules, we can generate human-readable explanations for better comprehension.

1. increases from time 70 to 71 with average value 0.79 ≤ 12.5 and decreases from time 23 to 29 with an average value -0.07 ≤ 3.5 ⇒ Myocardial Infarction
2. increases from time 70 to 71 with average value 0.93 > 12.5 and decreases from time 23 to 29 with an average value -0.07 < 3.5 ⇒ Normal Heartbeat
3. increases from time 70 to 71 with average value 0.79 > 12.5 ⇒ Normal Heartbeat

The objective evaluation results shed light on the effectiveness of our novel *post-hoc* XAI method in explaining the inference process of deep learning-based time series classification models.

The competitive accuracy results show the reliability of the decision trees generated to capture the essence of the underlying deep learning models. Fidelity, which measures how well the surrogate model predictions match the models' decisions, showed strong results for both types of models we tested. However, there is still a need for additional metrics to ensure the faithfulness of the generated explanation.

The robustness scores, especially for ECG200, FordA and FordB on both FCN and LSTM-FCN, indicate the resilience of the proposed XAI method in producing consistent and reliable explanations for insignificant changes in the data that do not affect the model prediction.

The decision tree graphs are relatively simple for smaller datasets with a low number of nodes and depth. However, for FordA and FordB, the number of depth and nodes, especially the standard deviation, is higher. This is primarily attributed to our automatic selection of the optimal alpha value for post-pruning the decision tree. Manual selection of the optimal alpha value, accounting for the number of nodes, depth, and accuracy, could have created a more interpretable decision tree graph.

These findings suggest that the proposed method can generate interpretable explanations using relatively simple decision trees that are easily understandable to users. The core strength of our methodology lies in its ability to avoid time series data segmentation, choosing instead the direct extraction and clustering of parameterized event primitives to provide rule-based global explanations. This approach not only simplifies the feature space but also ensures the faithful representation of temporal relationships within the time series in the resulting explanation model. Despite this, it is crucial to recognize a potential limitation concerning its performance on more complex datasets, especially those with higher dimensionality, such as multivariate time series. In such cases, the resulting decision tree graphs might become more intricate and pose interpretation challenges. However, the proposed method could be extended to address these challenges by incorporating more sophisticated clustering or feature extraction techniques.

## 6 Conclusion and future work

This paper introduced a novel model-agnostic XAI method for deep learning-based time series classification models. The proposed method utilizes a decision tree graph to show the crucial time steps in the model prediction. The study evaluated the explanation generated by this approach using various objective metrics such as accuracy, fidelity, depth, number of nodes and robustness. The findings of this research provide a strong foundation for developing more transparent and interpretable XAI methods for state-of-the-art deep learning models in the future. Our experiments suggest that the explanation becomes more interpretable with a reduced depth and number of nodes. Moving forward, we plan to validate this method on complex and multivariate time series datasets and conduct a human-centered evaluation of the explanations generated by this method in comparison to existing XAI methods for time series.

## Data Availability

The original contributions presented in the study are publicly available. This data can be found here: https://www.timeseriesclassification.com/.
