# Supplementary material for: A global model-agnostic rule-based XAI method based on Parameterized Event Primitives for time series classifiers
Source: Front Artif Intell. 2024 Sep 20;7:1381921. doi: 10.3389/frai.2024.1381921 (PMC11449859; doi:10.3389/frai.2024.1381921)
Supplement: Supplementary file 1 [file Data_Sheet_1.pdf]

Supplementary Material

1 DECISION-TREE GRAPHS PRODUCED BY THE METHOD

The presented figures below offer visual representations of decision tree graphs, meticulously crafted through our proposed methodology. These graphs provide a global decision tree-based explanation for the inference process of the models we tested (i.e. FCN and LSTM-FCN) across different datasets, including ECG, Gunpoint, Ford A, and Ford B.

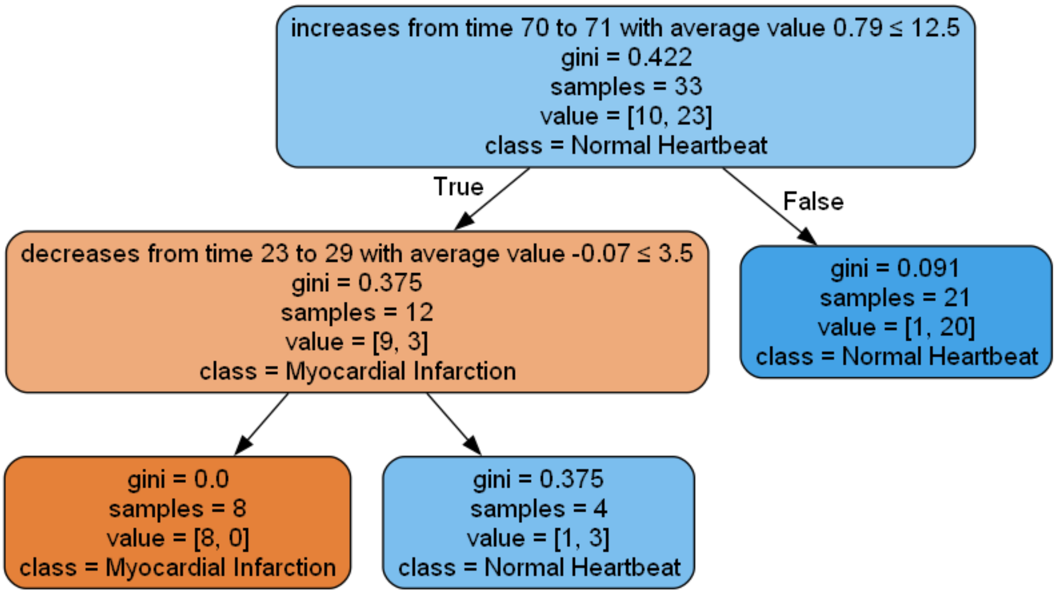

Figure S1: Visualization of decision tree graph produced by the proposed method applied to ECG data for the FCN model

2 OPTIMAL ALPHA (α) FOR DECISION TREE PRUNING

Figure S9 and S10 show the correlation between alpha values and decision tree accuracy, as well as the node and depth metrics across all four datasets. To guarantee the interpretability of our explanations, we utilize a post-pruning technique called Cost Complexity Pruning (CCP). In this experiment, the alpha is automatically selected based on the test accuracy tree. However, it is advisable to manually choose the optimal alpha value by considering factors such as the number of nodes, depth, and accuracy derived from the produced plots (see Figure S9 and S10). Subsequently, retraining the decision tree with the chosen alpha aims to achieve a balanced decision tree with fewer nodes and reduced depth, all while minimizing any potential impact on overall performance.

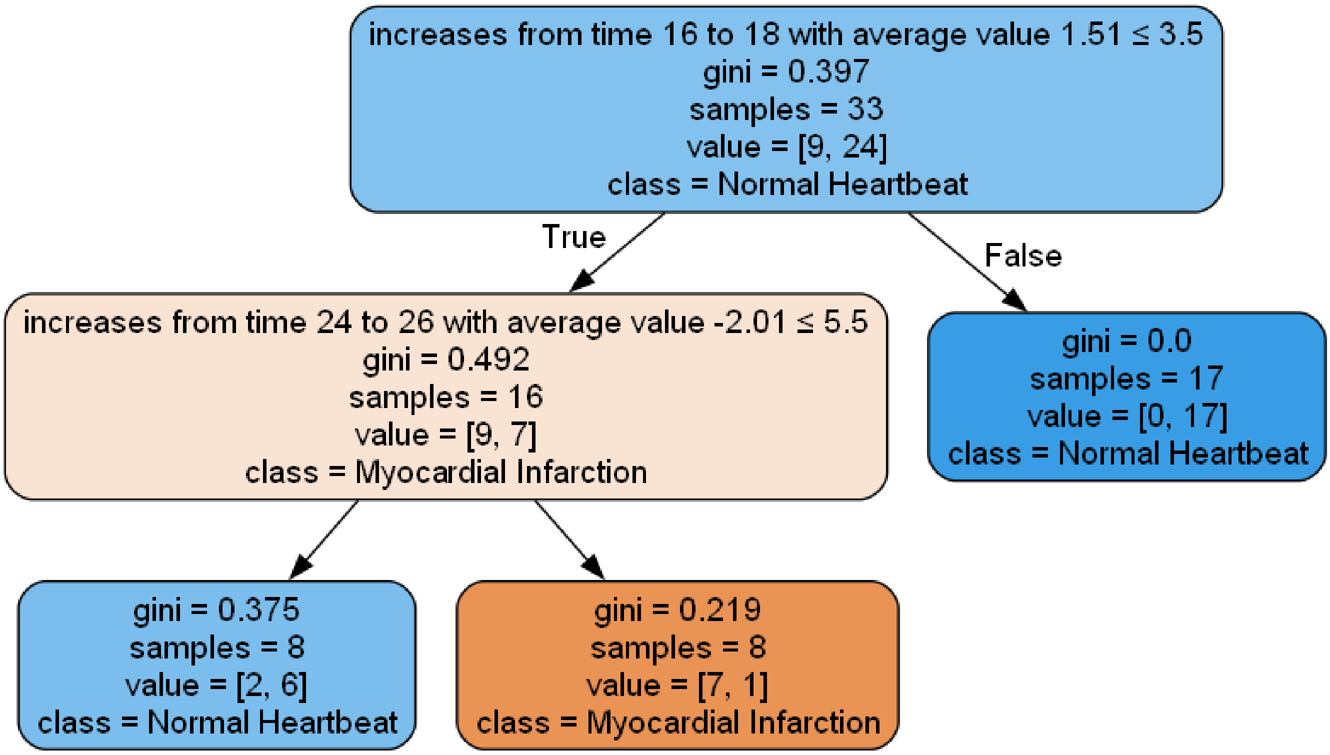

Figure S2: Visualization of decision tree graph produced by the proposed method applied to ECG data for the LSTM-FCN model

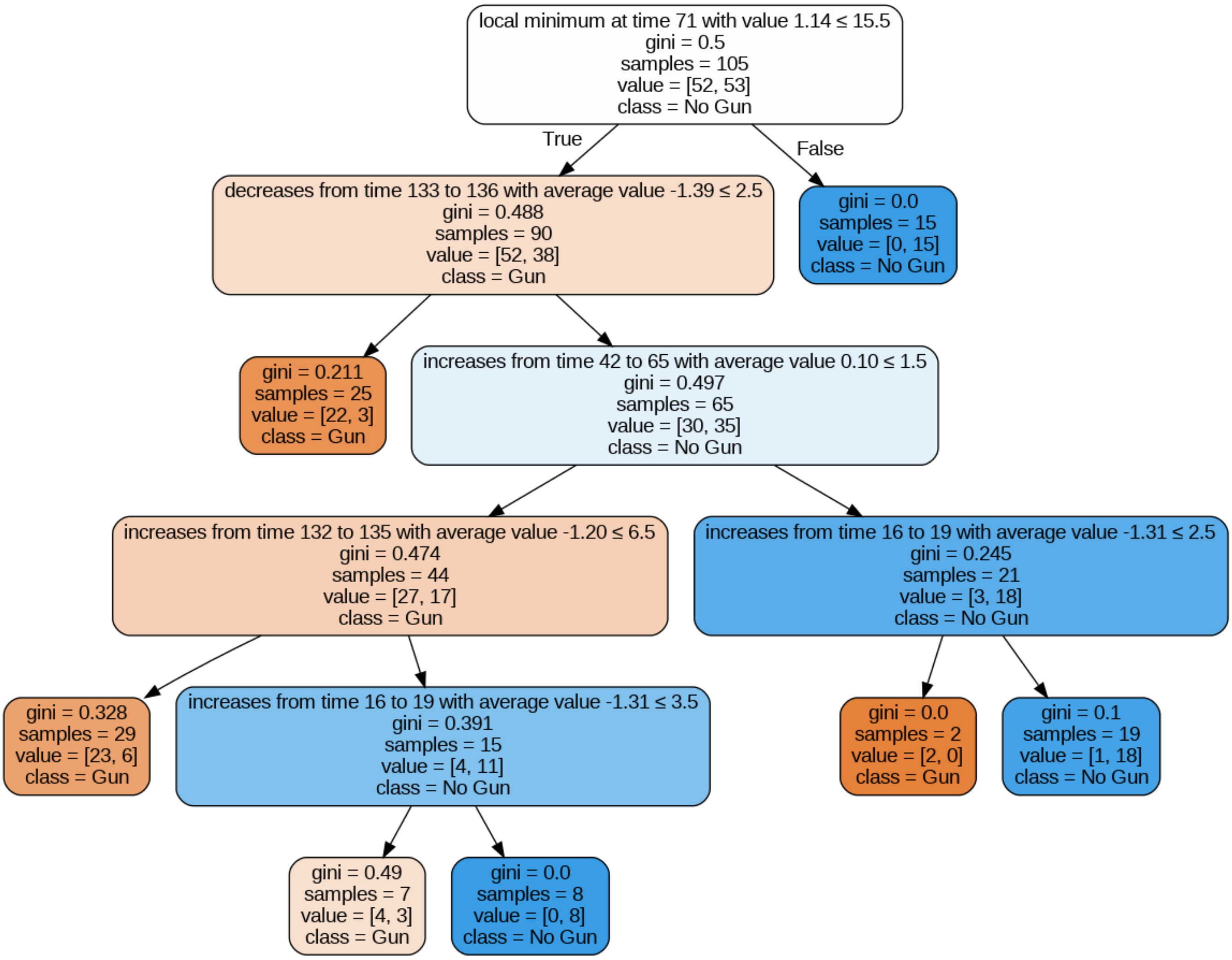

Figure S3: Visualization of decision tree graph produced by the proposed method applied to GunPoint for the FCN model

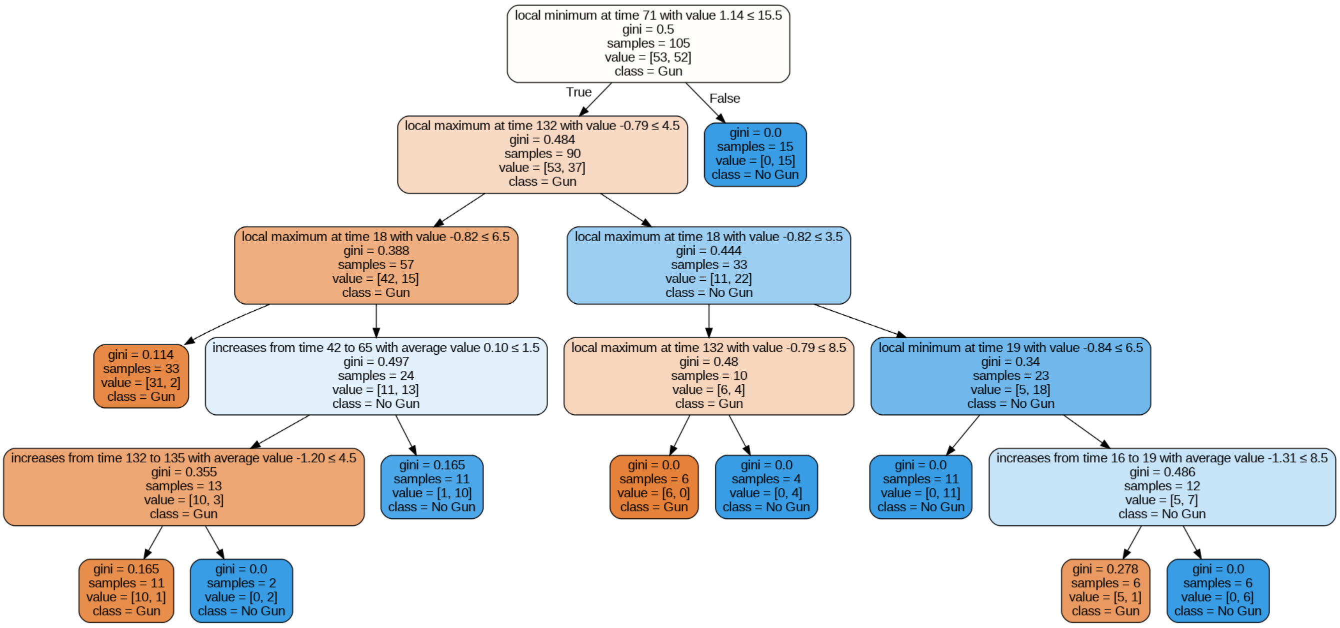

Figure S4: Visualization of decision tree graph produced by the proposed method applied to GunPoint data for the LSTM-FCN model

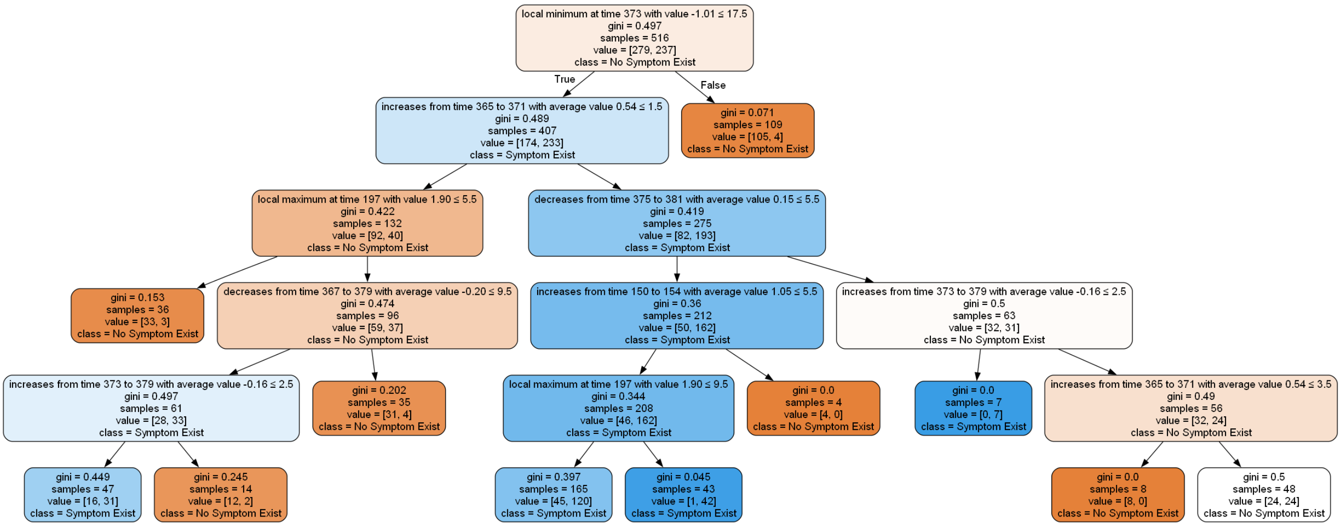

Figure S5: Visualization of decision tree graph produced by the proposed method applied to FordA for the FCN model

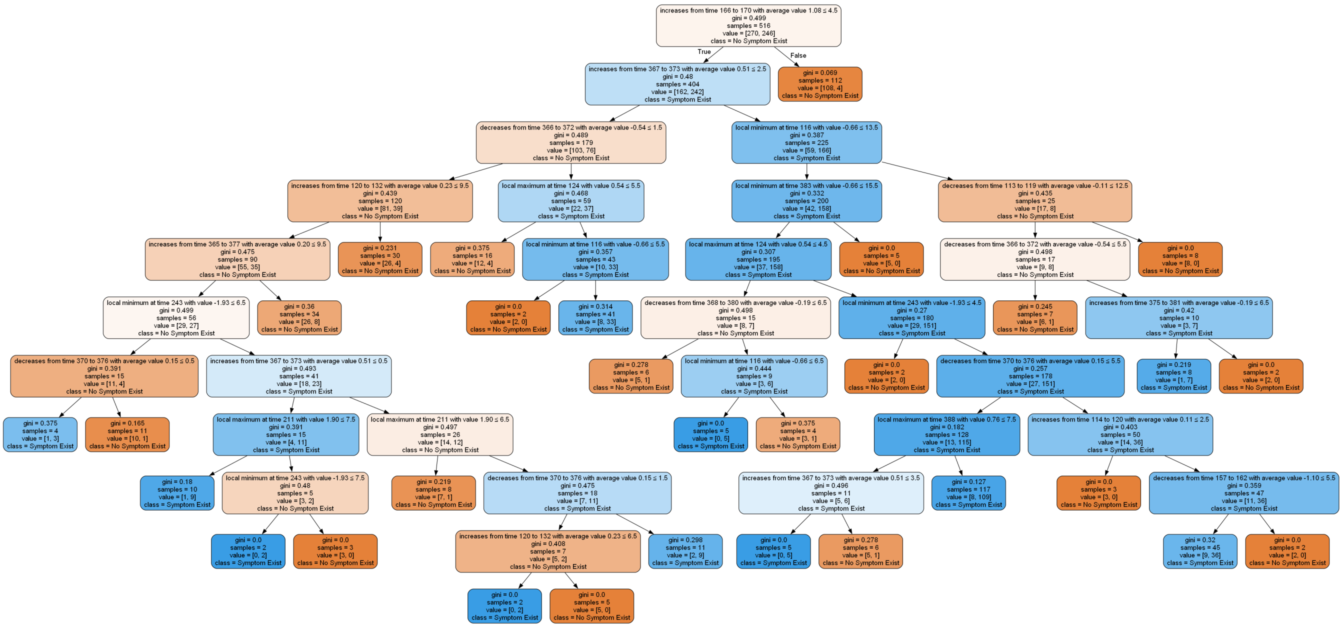

Figure S6: Visualization of decision tree graph produced by the proposed method applied to FordA data for the LSTM-FCN model

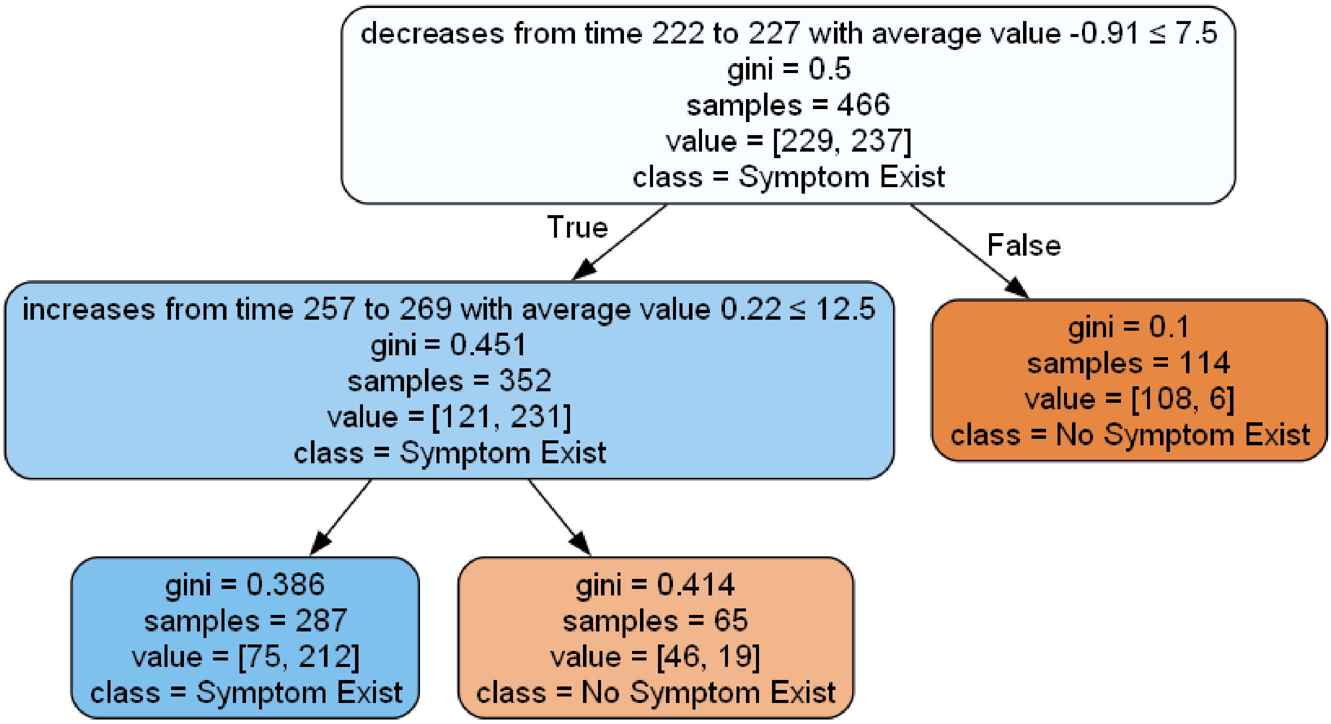

Figure S7: Visualization of decision tree graph produced by the proposed method applied to FordB for the FCN model

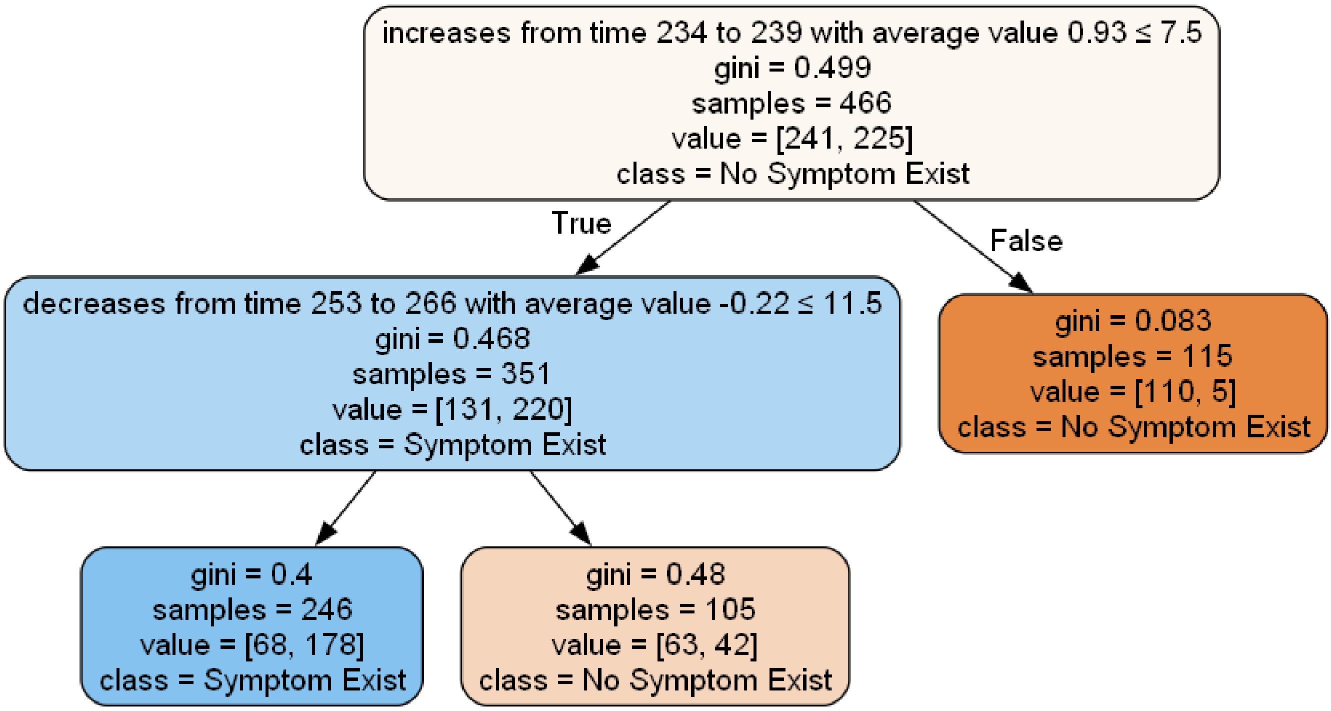

Figure S8: Visualization of decision tree graph produced by the proposed method applied to FordB data for the LSTM-FCN model

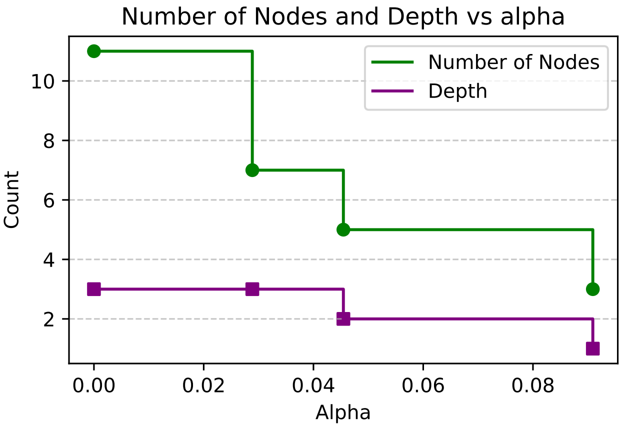

(a) Number of Nodes and Depth vs Alpha (ECG200)

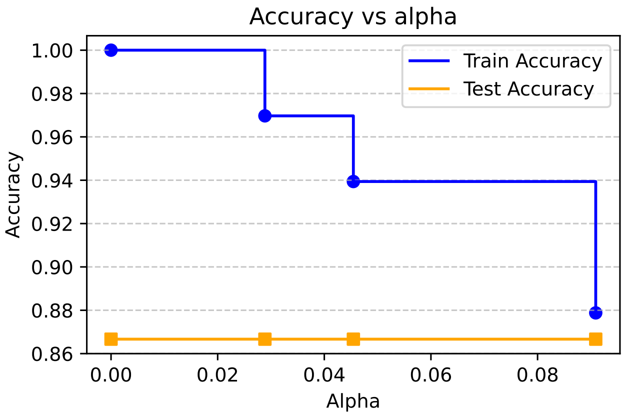

(b) Train/Test Accuracy vs Alpha (ECG200)

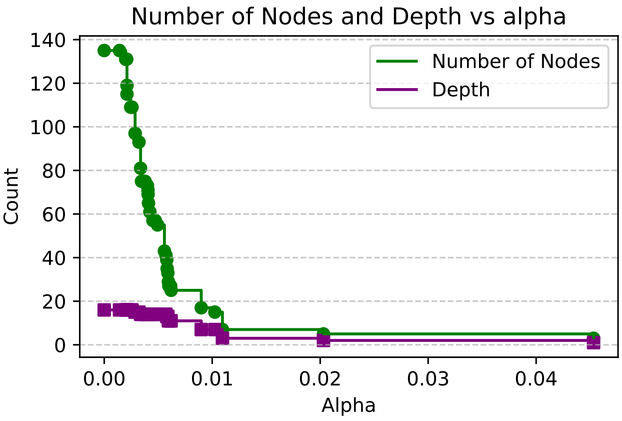

(c) Number of Nodes and Depth vs Alpha (Ford A)

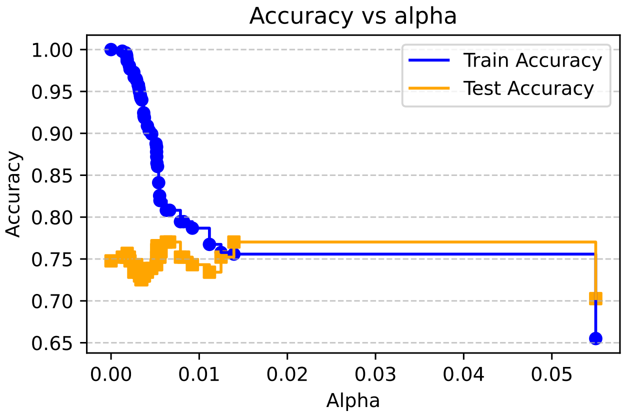

(d) Train/Test Accuracy vs Alpha (Ford A)

Figure S9: Plot of optimal alpha value for pruning decision tree for ECG200, and Ford A datasets

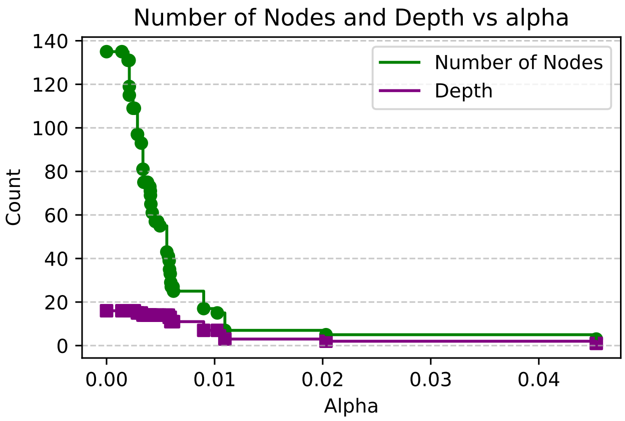

(a) Number of Nodes and Depth vs Alpha (Ford B)

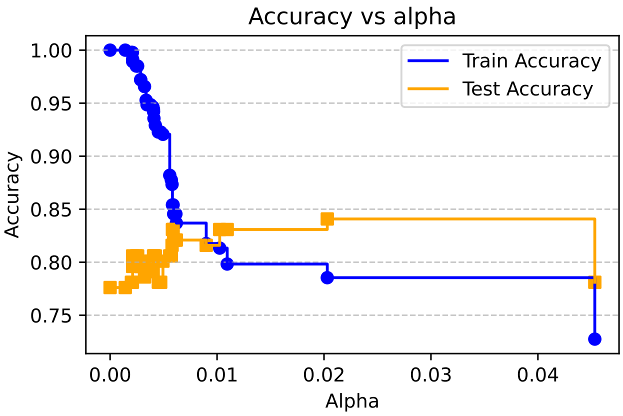

(b) Train/Test Accuracy vs Alpha (Ford B)

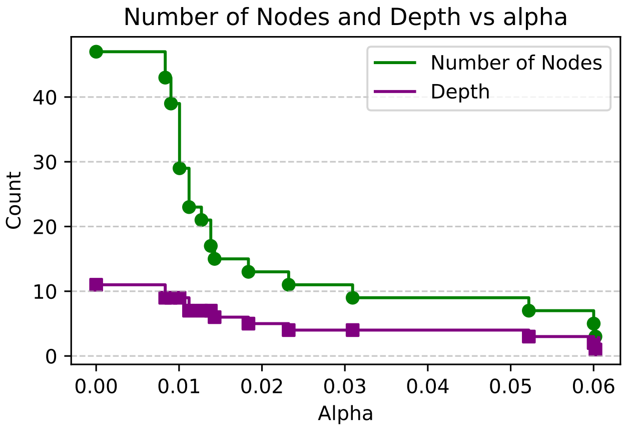

(c) Number of Nodes and Depth vs Alpha (GunPoint)

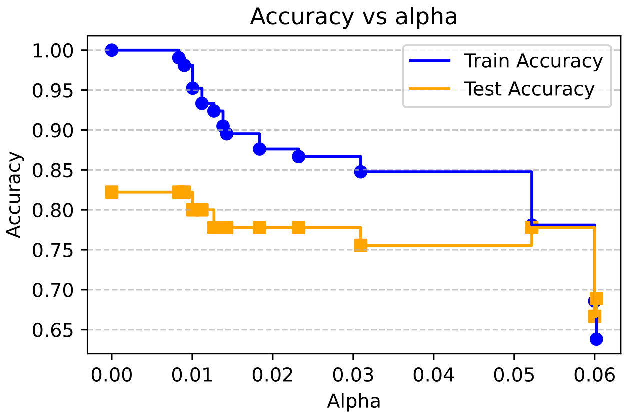

(d) Train/Test Accuracy vs Alpha (GunPoint)

Figure S10: Plot of optimal alpha value for pruning decision tree for Ford B and Gunpoint datasets
